# Capitated Telehealth Coaching Hospital Readmission Service in Australia: Pragmatic Controlled Evaluation

**DOI:** 10.2196/18046

**Published:** 2020-12-01

**Authors:** Carmel Martin, Narelle Hinkley, Keith Stockman, Donald Campbell

**Affiliations:** 1 Monash Health Faculty of Medicine Nursing and Health Sciences Monash University Clayton Australia; 2 Community Health Monash Health Dandenong, Victoria Australia; 3 Northern Health Northern Hospital Epping, Victoria Australia

**Keywords:** telehealth, funding model, evaluation, health services research, potentially preventable hospitalizations, medical informatics

## Abstract

**Background:**

MonashWatch is a telehealth public hospital outreach pilot service as a component of the Government of Victoria’s statewide redesign initiative called HealthLinks: Chronic Care. Rather than only paying for hospitalizations, projected funding is released earlier to hospitals to allow them to reduce hospitalization costs. MonashWatch introduced a web-based app, Patient Journey Record System, to assess the risk of the journeys of a cohort of patients identified as frequent admitters. Telecare guides call patients using the Patient Journey Record System to flag potential deterioration. Health coaches (nursing and allied health staff) triage risk and adapt care for individuals.

**Objective:**

The aim was a pragmatic controlled evaluation of the impact of MonashWatch on the primary outcome of bed days for acute nonsurgical admissions in the intention-to-treat group versus the usual care group. The secondary outcome was hospital admission rates. The net promoter score was used to gauge satisfaction.

**Methods:**

Patients were recruited into an intention-to-treat group, which included active telehealth and declined/lost/died groups, versus a systematically sampled (4:1) usual care group. A rolling sample of 250-300 active telehealth patients was maintained from December 23, 2016 to June 23, 2019. The outcome—mean bed days in intervention versus control—was adjusted using analysis of covariance for age, gender, admission type, and effective days active in MonashWatch. Time-series analysis tested for trends in change patterns.

**Results:**

MonashWatch recruited 1373 suitable patients who were allocated into the groups: usual care (n=293) and intention-to-treat (n=1080; active telehealth: 471/1080, 43.6%; declined: 485, 44.9%; lost to follow-up: 178 /1080, 10.7%; died: 8/1080, 0.7%). Admission frequency of intention-to-treat compared to that of the usual care group did not significantly improve (*P*=.05), with a small number of very frequent admitters in the intention-to-treat group. Age, MonashWatch effective days active, and treatment group independently predicted bed days. The analysis of covariance demonstrated a reduction in bed days of 1.14 (*P*<.001) in the intention-to-treat group compared with that in the usual care group, with 1236 bed days estimated savings. Both groups demonstrated regression-to-the-mean. The downward trend in improved bed days was significantly greater (*P*<.001) in the intention-to-treat group (Sen slope –406) than in the usual care group (Sen slope –104). The net promoter score was 95% in the active telehealth group compared with typical hospital scores of 77%.

**Conclusions:**

Clinically and statistically meaningful reductions in acute hospital bed days in the intention-to-treat group when compared to that of the usual care group were demonstrated (*P*<.001), although admission frequency was unchanged with more short stay admissions in the intention-to-treat group. Nonrandomized control selection was a limitation. Nonetheless, MonashWatch was successful in the context of the HealthLinks: Chronic Care capitation initiative and is expanding.

## Introduction

### Overview

Potentially preventable hospitalizations or potentially avoidable admission costs are of significant interest, not only to governments and hospitals, but to individuals, their families, the community, and general practice [1]. A pragmatic study evaluated the impact of MonashWatch, a telehealth coaching capitated pilot service in Victoria, Australia, on bed days in the context of a statewide rollout of a new funding model. Rather than only paying for hospitalizations, projected admission funding is released in advance to hospitals to allow them to develop systems that will reduce preventable hospitalizations.

HealthLinks: Chronic Care (HLCC) is a voluntary, funding-neutral reform that aims to support the Australian State of Victoria’s public health services in adopting outcome-based, rather than activity-based, funding [2]. An algorithm running on hospital data identifies patients at-risk of potentially preventable hospitalizations and informs participating hospitals who can use financing from anticipated admissions to address care needs better and earlier.

The MonashWatch telehealth and coaching model used design principles to establish a collaborative patient-journey approach responding to broad social determinants beyond disease management and the boundaries of hospital, primary, home, and social care.

Laypersons called *telecare guides* track risk and identify issues in biopsychosocial and environmental domains using frequent telephone calls and the Patient Journey Record System, which uses a client-server architecture with a browser-based user interface. A rule-based algorithm provides a real-time risk assessment of calls based on data entry and telecare guide opinion. *Health coaches* triage calls and support participants to optimize their health journeys.

This paper reports a pragmatic summative evaluation of the MonashWatch service. We compared bed days for an intention-to-treat group versus a usual care control group for 30 months from the MonashWatch service commencement. The intention-to-treat group included a MonashWatch active telehealth group consisting of those who used the telehealth service.

### Background

The Australia-wide universal free public health system has both federal and state/territorial governance. Parallel private health systems exist. Medicare is the Australian federal government’s scheme to give universal public access to health care (funded by taxation—the Medicare levy) through (1) direct clinical service funding to general practitioners and specialists in all states and territories and (2) indirect financing, with the states and territories administering public hospital and most community services.

Most services, including social services and welfare, aged care, education, and employment, have split funding and administration across federal and state/territory systems.

There have been multiple initiatives to address avoidable admission costs across the jurisdictions for more than 20 years. The Australian Institute of Health and Welfare (AIHW) annually reports on 18 International Statistical Classification of Diseases, Tenth Revision (ICD-10) diagnostic codes of chronic, acute, and vaccination-preventable admissions for national performance monitoring by local area [1]. Since 2000, there have been two significant waves of coordinated care trials which pooled federal and state resources (and sometimes private health resources) in several local trials to improve potentially preventable hospitalizations costs and health outcomes [3]. Significantly higher health service use and costs were incurred in the absence of clear evidence of improved health outcomes. Many clients did not require care coordination. Funds pooling arrangements contributed to limited possibilities for service substitution, and training of general practitioner care coordinators was inadequate [3]. Ambitious large-scale randomized controlled methods in health service transformation trials, which have failed due to the poor implementation of live services, have been part of the problem [3]. Ongoing and continually changing federal and state/territory integrated care initiatives have similarly failed to document improvements for care costs and outcomes [4]. Nevertheless, the Australian health system is high performing. In 2016, health expenditure as the proportion of gross domestic product was 9.6% for Australia, 9.0% for all Organization for Economic Co-operation and Development countries, 9.7% for the United Kingdom, and 10.6% for Canada; and Australia’s healthy life expectancy is 73 years, around 10 years higher than the global average life expectancy [5].

### Victorian Government Funding Reform Initiative in Acute Hospital Use

Victoria is the second most populous state of Australia with a population of 6.25 million. The Victorian Department of Health and Human Services (DHHS) funds and administers 85 public health service organizations. It has had activity-based funding (fee-for-service) for all acute admissions with accelerating demand growth in hospital separations per 1000 individuals. Victoria has deployed hospital readmission prevention programs since 1996, which have improved satisfaction with care but have had little impact on cost containment [6].

The HLCC initiative began in 2016, identifying patients at-risk of ≥3 repeat hospitalizations and their admission costs in the subsequent 12 months [7]. The DHHS incentivizes participating hospital systems to improve admission bed days, costs, and care quality within projected costs (HLCC-identified patients).

### Monash Health

Monash Health is the most extensive public hospital and community care system in Victoria. Its 15,000 staff (with a large hospital readmission prevention program base) work at more than 40 sites, providing more than 3 million occasions of service, admitting more than 238,000 hospital patients, and handling more than 206,000 emergency presentations per year. HLCC data indicated that in 2017 and 2018, Monash Health had more than 3000 patients with >4 acute medical admissions and more than 12,000 with >3 acute medical admissions (30% of which were potentially preventable hospitalizations). It was an early adopter of the HLCC service initiative MonashWatch in 2016.

Rather than adding a new layer, the MonashWatch model was intended to be a catalyst within a working health system, to begin a transition of acute services to outcome-based funding.

### MonashWatch Model of Care

Patient journeys involve physiological, psychological, social, and environmental issues. Disturbances in any or combinations of domains including housing, food security, support with daily living, access issues, as well as biology, medication issues, or clinical deterioration may lead to tipping points into acute admissions [8,9]. Therefore, MonashWatch focused on a journey model to enable individuals to optimize their health trajectory with their caregivers and essential people, rather than focus mainly on their selected diseases and treatment adherence [10]. The Patient Journey Record System was designed to monitor health journeys and was initially developed in Ireland and validated in an Irish primary care cohort [11,12]. A key feature is that telecare guides, who are nonprofessionals, engage with people from their community to monitor and support their care. The structure of the Patient Journey Record online system and the supervision of coaches ensure the quality and safety of telecare guide work.

Clinicians—nursing or allied health—enable others in the goals and health journeys which they chose to follow [13]. Coaching, in the MonashWatch context, is an innovative flexible transdisciplinary role. The MonashWatch coaching role, incorporating the Patient Journey Record System journey model, empowers clinicians from multiple disciplines to bring their specific expertise and collaborate across disciplinary boundaries to enable person-centered rather than professional-centered siloed care. The role was developed through extensive consultation with patients and advocacy groups, service providers, and the DHHS. The role of the coach providing oversight and support for telecare guides, as well as individual patient coaching, is essential. The advantage of a multidisciplinary team role is the very broad scope of integrated practice—nursing, physiotherapy, social work, occupational therapy, and medicine. A coaching framework was constructed to address health, resilience, and need perceptions rather than protocol-driven care and to guide people through a health and welfare maze. Coaches use HLCC-capitated funds for food, transport to outpatients, or general practitioners, or a second opinion in the private sector as needed.

### Active Telehealth Service

Following HLCC algorithm identification and allocation, the intention-to-treat group were invited to initially consent and agree to a home visit for enrollment, formal consent, baseline assessment, and induction. Telecare guides, then made conversational phone calls to enrolled people to track their health and needs, in accordance with personal preference and previous Patient Journey Record System flags.

**Figure 1:**
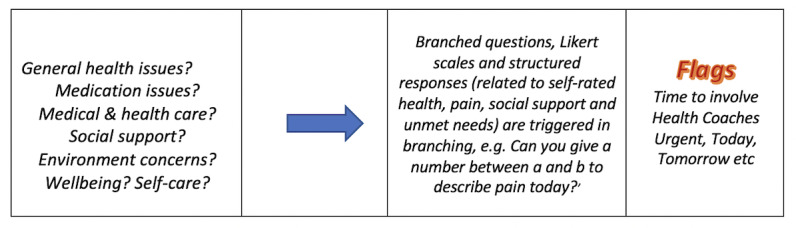
Patient Journey Record semistructured monitoring app that begins with open-ended narratives and includes direct questions.

**Figure 2:**
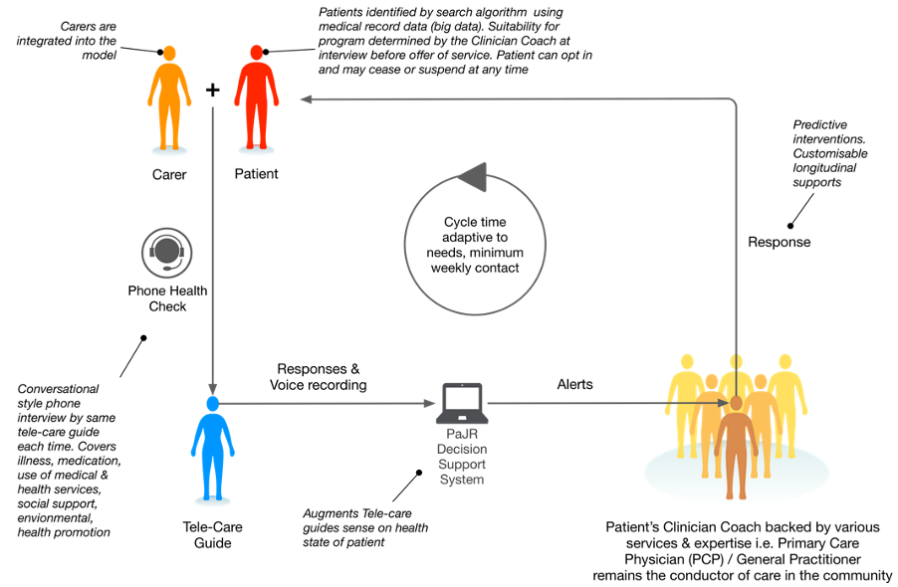
The MonashWatch model (reproduced from Martin et al [14]). PaJR: Patient Journey Record.

Regular audiotaped calls between 1 to 5 times per week (median 1), depending on risk level, were conducted by telecare guides. They used the Patient Journey Record System semistructured monitoring app, which began with open-ended narratives and included directed questions [11] in a branching format (Figure 1) Patient Journey Record System flags were traffic-light indicators of physical and psychosocial resilience and symptomatology generated from the conversations in real time using an internal algorithm [11,12]. Flags implicated risk fluctuations in short-term intervals (hours to days) in individual journeys that may foreshadow potentially preventable hospitalizations admissions. Patient Journey Record System risk analysis guided telecare guide decision making on the timeframe (eg, immediately, tomorrow, etc) of the need to involve their health coaches. Telecare guides and coaches called the same individuals and got to know them over time, forming relationships. Coaches triaged calls and intervened to address urgent or nonurgent and high, medium, and lower risk issues promptly, including the needs of carers. Coaches worked directly with the general practitioner who was the primary medical provider. Coaches also worked directly with emergency departments; inpatients; outpatients; and drug, alcohol, and social services on an as-needed basis. Coaches triaged and anticipated risk of health deterioration using the PaJR predictive algorithm and human sensemaking [14]. They, then, identified and intervened in the root causes of readmissions, where possible [14] (see also Figure 2). Coaches triaged, navigated, and supported MonashWatch participants in a reactive and anticipatory manner bridging the gaps with general practitioner, hospital, pharmacy, social welfare, housing, legal, and other support services. Coaches made telephone contact, home visits, and accompanied participants to general practitioners, clinics, and other facilities, where appropriate.

## Methods

### Implementation of the MonashWatch Model

The health coaches were all recruited from within the Monash Health community and acute services to develop the new service. Telecare guides were recruited from the local or adjacent community. Care pathways to existing services were mapped and tested before the service commenced. The pathways were to the general practitioners, hospitals’ emergency, inpatient and outpatients, hospital readmission prevention programs, and other community health, social, and welfare services including housing, legal, financial, employment, education, and voluntary organizations.

### Participants

MonashWatch participants were selected in a highly disadvantaged catchment area adjacent to the Dandenong hospital. Keeping the MonashWatch team local to patients was a concept borrowed from Buurtzorg: the Dutch neighborhood care model [15]. Targeted patients resided within a short car trip from the MonashWatch team location to minimize clinician travel time and hence maximize responsiveness and clinical time. Most admissions for patients in the cohort occurred at the local public secondary care hospital in Dandenong. However, some took place in other more distant Monash Health hospitals, subject primarily to demand and clinical needs.

### Potentially Preventable Hospitalizations

The HLCC web-based algorithm incorporated a wide range of conditions in adults >18 years old [2]. The HLCC algorithm incorporated the following: (1) service parameters including rates of recent acute admissions and emergency department visits; (2) patient parameters including age, residence status, smoking; (3) chronic conditions such as gastrointestinal disorders, renal disease, asthma, chronic obstructive pulmonary disease, rheumatoid arthritis, diabetes, pancreatic conditions, cirrhosis/alcoholic hepatitis; but excluded serious mental and psychotic illnesses, dialysis, and cancer treatments because there are other initiatives for these groups.

AIHW potentially preventable hospitalizations diagnostic codes only accounted for 18% of HLCC admissions. Only approximately 20% of HLCC-identified hospitalizations in Monash Health, and other Victorian health services had ever accessed hospital readmission prevention programs or other hospital admission prevention services.

### Participant Enrollment Allocation

The DHHS provides continuously updated HLCC-eligible cohort lists to hospital groups and funds care improvement initiatives based on projected reductions in admission costs. Once the patient was deemed eligible, when they had their next acute admission, they could be enrolled. Enrollment commenced with a gradual ramp-up from December 2016 and continued beyond the evaluation cutoff point. Pragmatic screening by health coaches excluded those who were not suited to a self-rated phone-based health model (eg, nursing home, necessitated use of an interpreter, and patients who would pose a high risk to staff visiting at home). Patients were considered candidates to be entered into the MonashWatch evaluation pool before allocation, based on a ratio of 4:1. There was minimal chance of bias because the health coaches and team performing the assignment had no idea who would benefit in advance in this pilot service, and the allocation was conducted using hospital unit numbers from a list without patient details.

### Outcomes

The primary outcome metric was bed days (ie, length of stay related to emergency nonsurgical admissions) derived from the Victorian Admitted Episode Data from the Victorian Emergency Minimum Dataset [16] between December 23, 2016 and June 23, 2019.

A secondary outcome metric was rate of emergency nonsurgical admissions. This was initially considered as the primary outcome; however, capitation costs being the biggest driver of the HLCC program led to bed days being more critical. Net promoter score was also a secondary outcome.

### Statistical and Other Methods

Analysis of covariance (ANCOVA) is a statistical technique that adjusts for covariates in determining the outcomes of an intervention. Least square means are an acceptable method to calculate the means adjusted for covariates [17]. In ANCOVA, least square means are group-mean adjusted for covariates (ie, holding constant at some typical value of the covariate, such as its mean value). Effective days active describes the duration in days post assignment to intention-to-treat or usual care. The outcome was least square of effective days active of control versus intervention, mean-adjusted for the quantitative variables above. Two-tailed Mann-Kendall trend test and Sen slope were used for the significance of the time series trend and to calculate an estimate of the trend. The net promoter score survey was anonymously administered (as a postal survey) with open-ended comments. The score was calculated following the Australian National Safety and Quality Health Service Standard [18]. The net promoter score is an index ranging from –100 to 100 that measures the willingness of customers to recommend a company's products or services to others. It is calculated as the difference between the percentage of promoters and detractors and is used as a proxy for gauging the patient’s overall satisfaction with a hospital’s product or service and the patient’s loyalty to the service [18]. Other secondary outcomes measures including baseline and sequential measures have previously been described [14]. A significance level of *P*<.05 was used. Analysis of bed days was conducted using least square ANCOVA, in accordance with acceptable practice [19]. Mean bed day values were adjusted for age, gender, time in the MonashWatch intervention (effective days active), and the presence of potentially preventable hospitalization—18 selected ICD-10 diagnostic codes—covariates [1].

Quality assurance of the data analysis was performed in several ways. One author conducted the ANCOVA using XLSTAT software (version 2020:4.1; Addinsoft). Two other authors independently analyzed the same dataset, after rechecking the download from Victorian Admitted Episode Data/Victorian Emergency Minimum Dataset [16], using R (version 3.5.2; R Studio). External evaluation was carried out on bed days and satisfaction with external controls using propensity-scoring rather than contemporaneous local controls and has not yet been formally reported.

Ethics approval for low-risk clinical research was obtained from the Monash Health's Health Research Ethics Committee. The Australian government’s main research and development agency, the Commonwealth Scientific and Industrial Research Organisation, is conducting an external evaluation of the diverse state-wide HLCC initiatives in Victoria, and this also has ethics approval.

## Results

### Implementation of the MonashWatch Model

The HLCC clinical algorithm identified 2502 patients as having a high risk of repeat admissions within the period of December 23, 2016 to June 23, 2019.

### Participants

**Figure 3:**
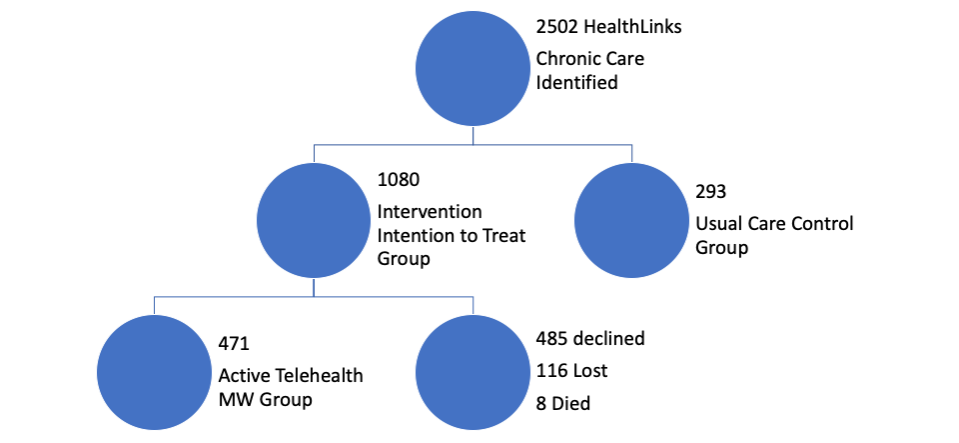
MonashWatch pragmatic clinical evaluation participants. Intention-to-treat, including active telehealth, and usual care cohort allocation in the MonashWatch pragmatic evaluation. MW: MonashWatch.

MonashWatch identified 1373 suitable HLCC patients: usual care (n=293) and intention-to-treat (all: n=1080; active telehealth: 471/1080, 43.6%; declined: 485/1080, 44.9%; lost to follow-up: 116/1080, 10.7%; died: 8/1080, 0.7%; Figure 3). The intention-to-treat active telehealth group provided consent to being recruited, and general practitioners, health, and social services were aware of their pilot service status—blinding was not possible. Controls had no contact with the MonashWatch team, and general practitioners, health, and social services were unaware that they were controls. Once allocated to the intention-to-treat and the active telehealth groups, patients remained in that group for the duration.

Mean participant age for usual care was 64.3 (SD 17.6; median 71, IQR 19) years, and mean participant age for intention-to-treat was 68.3 (SD 16.8; median 71, IQR 19) years. The number of effective days active for usual care was 756.2 (SD 180.5; median 1003, IQR 44), and the number of effective days active for intention-to-treat was 624.2 (SD 269.2; median 908, IQR 278).

### Admissions

In the usual care group, 293 patients had 639 admissions, and in the intention-to-treat group, 1080 had 934 admissions from the time they joined the MonashWatch program (effective days active) until June 23, 2019 (Table 1). Usual care had 163/293 (55.6%) patients admitted at least once; a median of 3 admissions/person who was admitted (third quartile 5); a mean of 3.9 admissions/person who was admitted (SD 4.3, skewness 3.1). Intention-to-treat had 549 (50%) of patients admitted at least once; a median of 2 admissions/person who was admitted (third quartile 4); a mean of 3.6 admissions/person who was admitted (SD 4.3, skewness 6.8). Men were 44.2% (72/163) and women were 55.8% (91/163) of admissions in usual care. Men were 55.9% (307/549) and women were 44.1% (242/549) of admissions in intention-to-treat.

**Table 1:** Descriptive statistics on admission rates in MonashWatch intention-to-treat and usual care.

Statistic		Usual care	Intervention	*P* value
Admitted at least once, n (%)		163 (55.6)	549 (50.8)	.054
**Description of profiles of those admitted in each group**				
	Median (third quartile)	3 (5)	2 (4)	.05
	Mean (sample SD)	3.9 (4.3)	3.6 (4.6)	.056
	Pearson skewness	3.1	6.8	<.001

Admissions were highly skewed in the intervention group with several outliers with frequent short admissions to the emergency department for chest pain and abdominal symptoms which may account for the highly skewed profile of the intention-to-treat group. The raw median and mean number of admissions per person were higher in the control group (*P*=.05 and *P*=.056, respectively; see Table 1).

### Potentially Preventable Hospital Admissions

AIHW potentially preventable hospital admission codes were present in 18.3 % (117/639) of all acute admissions in usual care and 16.4% (153/ 934) of all acute admissions in intention-to-treat (*P*=.34), that is, 0.05% (8/1573) of all admissions were coded vaccine preventable. The most frequent AIHW potentially preventable hospitalizations conditions were chronic obstructive pulmonary disease or bronchiectasis (usual care: 30/641, 4.7%; intention-to-treat: 49/934, 5.2%; *P*=.19); heart failure or angina (usual care 17/641, 2.7%; intention-to-treat 24/934, 2.6%), urinary tract infection or cellulitis (usual care 32/642, 5.6%; intention-to-treat 30/934, 3.2%; *P*=.04). Chest pain (of minor complexity) was the most frequent admission description in all groups (Table 2). Two individuals, who called an ambulance for chest pain (of minor complexity) at least weekly and had frequent <1-day overnight stay admissions to the emergency department, were outliers in the MonashWatch active telehealth group. The nonactive telehealth group had fewer diagnoses of chest pain (of minor complexity), abdominal pain and mesenteric adenitis, minor complexity and other digestive system disorders, and minor complexity diagnoses than the intention-to-treat group. Low complexity conditions including chest pain and gastrointestinal conditions were coded (usual care: 86/641, 20.4%; intention-to-treat: 219/1784, 12.2%). The usual care group had significantly more minor complex conditions diagnoses than the intention-to-treat group (*P*=.03). Diagnoses using AIHW potentially preventable hospitalization ICD codes that were designated as major complexity (high cost and high comorbidity) [20] are indicated. In the top 10 most frequent potentially preventable hospitalizations (Table 2), the usual care group had 3.0% (19/641) of admissions designated as major complexity, MonashWatch intention-to-treat active telehealth participants had 5.1% (48/934) of diagnoses identified as major complexity—significantly more than usual care (*P*=.02), and intention-to-treat nontelehealth participants had 1.1% (9/852) (*P*<.001).

**Table 2:** Most common diagnoses for potentially preventable hospitalizations as defined by the AIHW within each group of MonashWatch patients.

Group and diagnosis		Admission, n (%)
**Control (usual care, n=641)**		
	Chest pain, minor complexity	46 (7.2)
	Abdominal pain and mesenteric adenitis, minor complexity	20 (3.1)
	Other digestive system disorders, major complexity	19 (3.0)
	Bronchitis and asthma, minor complexity	18 (2.8)
	Esophagitis and gastroenteritis, minor complexity	18 (2.8)
	Arrhythmia, cardiac arrest and conduction disorders, minor complexity	16 (2.5)
	Other digestive system disorders, minor complexity	16 (2.5)
	Kidney and urinary tract infections, minor complexity	15 (2.3)
	Syncope and collapse, minor complexity	15 (2.3)
	Chronic obstructive airways disease, minor complexity	14 (2.2)
**Intention-to-treat including active telehealth (n=934)**		
	Chest pain, minor complexity	110 (11.8)
	Abdominal pain and mesenteric adenitis, minor complexity	41 (4.4)
	Respiratory infections and inflammations (major complexity	29 (3.1)
	Chronic obstructive airways disease, minor complexity	25 (2.7)
	Syncope and collapse, minor complexity	22 (2.4)
	Other digestive system disorders, minor complexity	21 (2.2)
	Other digestive system disorders, major complexity	19 (2.0)
	Headaches, minor complexity	18 (1.9)
	Esophagitis and gastroenteritis, minor complexity	18 (1.9)
	Arrhythmia, cardiac arrest and conduction disorders, minor complexity	16 (1.7)
**Intervention (intention-to-treat) nontelehealth (n=390)**		
	Chest pain, minor complexity	41 (10.5)
	Diabetes, minor complexity	10 (2.6)
	Arrhythmia, cardiac arrest and conduction disorders, minor complexity	9 (2.3)
	Abdominal pain and mesenteric adenitis, minor complexity	6 (1.5)
	Chronic obstructive airways disease, minor complexity	6 (1.5)
	Coronary atherosclerosis, minor complexity	6 (1.5)
	Poisoning/toxic effects of drugs and other substances, minor complexity	6 (1.5)
	Chronic obstructive airways disease, major complexity	5 (1.3)
	Syncope and collapse, minor complexity	5 (1.3)
	Heart failure and shock, major complexity	4 (1.0)

### Primary Outcome

ANCOVA was conducted on bed days with admission age, gender, the presence or absence of a potentially preventable hospitalizations ICD-10 code, and effective days active as quantitative variables and with intervention versus control as qualitative variables

**Figure 4:**
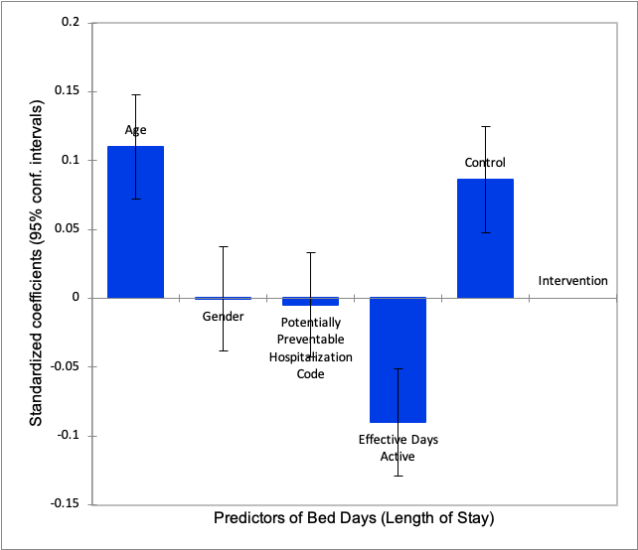
Bed days standardized coefficients based upon ANCOVA.

Table 3 and Figure 4 demonstrate the factors which significantly impacted on bed days in the sample: age (*P*<.001), effective days active from allocation until June 23, 2019 (*P*<.001), and whether they were in the intervention (intention-to-treat) or control (usual care) group (*P*<.001). Gender (*P*=.98) and having a designated AIHW potentially preventable hospitalizations admission ICD code (*P*=.82) were not significant predictors of bed days.

**Table 3:** ANCOVA summary statistics of the impact of key variables on bed days of MonashWatch patients: standardized coefficients predicting length of stay.

Source	Value	SE	*t* value	*P* value	95% CI
Admission age	0.110	0.019	5.673	<.001	(0.072, 0.148)
Gender	–0.001	0.019	–0.031	.98	(–0.038, 0.037)
AIHW^a^ potentially preventable hospitalizations (0, no; 1, yes)	–0.005	0.019	–0.235	.82	(–0.042, 0.033)
Effective days active	–0.090	0.020	–4.566	<.001	(–0.129, –0.051)
Control (usual care) vs intervention (intention-to-treat)	0.086	0.020	4.348	<.001	(0.047, 0.125)

^a^AIHW: Australian Institute of Health and Welfare.

Age, MonashWatch effective days active, and intention-to-treat group status predicted bed days. The usual care least square mean was 4.5 (SD 0.2, 95% CI 4.1-4.9) bed days while the intention-to-treat least square mean was 3.4 (SD 0.1, 95% CI 3.1-3.6) bed days. A statistically significant (*P*<.001) bed days saving of 1.14 bed days per 1080 intention-to-treat patients (1236 days) was estimated (Table 4).

**Table 4:** ANCOVA least square mean bed days comparison for usual care versus intention-to-treat groups.

Category	Mean bed days	SE	95% CI	*P* value
Usual care	4.5	0.2	(4.1, 4.9)	<.001
Intention-to-treat	3.4	0.1	(3.1, 3.6)	

### Usual Care Versus Intention-to-Treat

**Figure 5:**
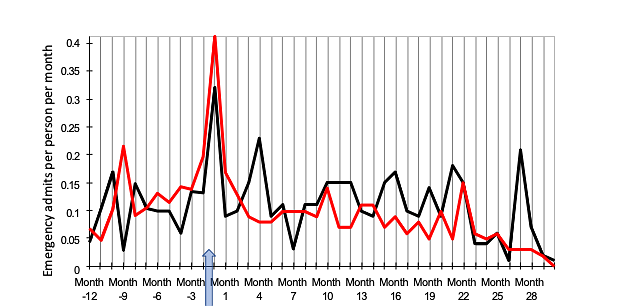
Rate of mean bed days per person per month in the usual care group (black) and intention-to-treat (red) 12 months prior to MonashWatch enrolment (indicated by the blue arrow) and for the subsequent 30 months. (Total admissions adjusted for numbers in each group.).

Longitudinal tracking of average bed days per person per month nonsurgical acute admissions was conducted for 12 months before enrollment until the evaluation cutoff date (Figure 5). The Mann-Kendall trend and Sen slope for intention-to-treat versus usual care demonstrated a significantly different trend in the intention-to-treat versus usual care time series. Rates of bed days per person per month in the 12 months before MonashWatch were higher in the intention-to-treat (1.77 bed days per person per month) versus usual care (1.44 bed days per person per month). Overall, intention-to-treat demonstrated statistically significant greater improvement (*P*<.001) in bed days per person per month (Kendall tau 0, sample variance 8483) compared with usual care (Kendall tau 0, sample variance 8458). The bed days per person per month time series demonstrated regression to the mean in both intention-to-treat and usual care groups. The intention-to-treat group demonstrated a statistically significant greater (*P*<.001) downward trend in bed days compared with the usual care group using Mann-Kendall trend test, and the Sen slope was –406 for intention-to-treat and the slope was –104 for usual care.

A high net-promoter rate (satisfaction scores) of 95% was demonstrated, with common findings of about 77% in hospital evaluations [18].

## Discussion

### Principal Findings

This evaluation demonstrates that the MonashWatch service intervention achieved its objectives by reducing bed days and, by implication, worked within the capitated budget consistently over time.

The percentage of patients admitted at least once was very similar (usual care: 55.6%; intention-to-treat: 50.8%). Admissions per person were nearly statistically significantly different (median, *P*=.05; mean, *P*=.056) with a frequent admitter intention-to-treat subgroup. The number of low complexity conditions, including chest pain and gastrointestinal conditions, was higher in the usual care group than it was in the intention-to-treat group. The intention-to-treat group had nonsignificantly more potentially preventable hospitalizations admissions, perhaps suggesting an optimization of hospital bed use, reducing admissions that were not related to clinical need alone. Analysis of covariance, controlling for age, effective days active, gender, and AIHW potentially preventable hospitalizations diagnoses demonstrated that bed days were statistically improved by 1.14 bed days per 1080 intention-to-treat patients (*P*<.001) and an estimated 1236 bed days. Improved bed days were consistent over the evaluation period. The intention-to-treat group demonstrated a consistent improvement in bed days per person per month (*P*<.001).

Challenges to constrain costs while improving care are prominent in the fragmented governance and funding system of Australia. Macrolevel federal and state reforms with pooled funding have not previously proven successful. Victoria has deployed state-based local initiatives to improve hospital readmissions since before 1996 but had identified the need to further “shift the dial [3,5,6].” A capitated funding model for hospital services to use projected admission costs earlier in a high admitting cohort was introduced in 2016 [2]. Monash Health took up this opportunity with unique internet-enabled telehealth (telephone) and coaching approach with real-time data usage. Using principles of disruptive innovation [21], MonashWatch formed an innovation hub within existing services and is becoming increasingly embedded in routine service delivery through its expansion and integration within other community services. Ongoing data-driven continuous improvement based on frequent audits and adaptation in the complex changing world of patient profiles and service changes will be a crucial feature [2].

### Did the Study Findings Demonstrate Causal Associations?

The tracking of bed days and triangulation with other findings indicated a causal association between MonashWatch and the improvement of bed day utilization using Bradford Hills criteria [22]. The effect size was 1.14 bed days saved per person, yet only 43.6% of the intention-to-treat group (471/1080) participated in the intervention suggesting a strong effect of the intervention. Positive effects using the Patient Journey Record System component of the MonashWatch model were demonstrated in 3 Irish cohorts [11] and another Victorian location [23]. MonashWatch has been judged by the Victorian DHHS to have improved efficiencies, with better care and external evaluation supporting a significant improvement in bed days of around 0.8 days per person [20]. Other evidence of a causal relationship is that a more significant reduction in bed days occurred immediately after entry into MonashWatch by the intention-to-treat group and persisted throughout the evaluation.

### Why Did the Intervention Appear to Work?

A plausible mechanism for the intervention working is addressing resilience and frailty through anticipatory care and coaching enabling stronger health networks and connections with vulnerable people. The results support a continuous adaptation to complex unstable health journey model for individuals [24]. Many hospital admissions, even very short stays in the emergency department (which are included in this study as an admission if there is an overnight stay) may be related to a wide range of indirect influences [25]. Underlying issues could include personality types, mood, anxiety or demoralization, drug and alcohol or medication management problems, service access issues and convenience, as well as the likelihood of benefit from hospital-level care [26,27]. A sense of discrimination has been described in emergency department frequent users [27]. Many of these factors (but not all) can be addressed through closer monitoring in a prehospital phase [28]. It is worth noting that a high net-promoter rate (satisfaction scores) of 95% was consistently reported by patients participating in MonashWatch at 6-month intervals and on exit. The accompanying narrative commentary was very positive about feeling “accepted” and “supported.” This commentary will be reported elsewhere and makes frequent mention of improved health experience and a sense that the hospital “cares.” This also suggests we are heading in the right direction.

The capitated structure provided very adaptable funding as needed for many issues such as transport, outpatient attendances, and home factors which underpin admissions. It provided coaches with the flexibility to go outside of health siloes working with general practitioners, hospital, alcohol, and mental health services.

Finally, this DHHS approach that is outcome and data-driven with continuous performance and costing review for teams and local initiatives keeps services from falling into complacency.

### Limitations

There is a range of limitations. The intention-to-treat group included 44.9% (485/1080) who declined. This arduous process may have diluted the uptake rate and thus had an impact on bed days but reflects real-world clinical service evaluations. It would be worth other methods of recruitment in the future to see if there would be increased recruitment with a more significant impact.

Pragmatic clinical evaluations in live health systems outside of research study are challenging, particularly in MonashWatch due to long-term unpredictable, complex dynamics in unstable patient journeys [14]. There are practical difficulties in finding true controls beyond the HLCC scoring algorithm. Systematic contemporaneous and ecological selection within a local geographical zone, as in this case, was the option judged to be most reliable. Retrospective propensity scoring based on HLCC, further stratified by diagnoses, age, and socioeconomic (postal code) status with randomly selected multiple controls in different hospitals, was employed by the external evaluation [20]. However, the findings of the MonashWatch approach were mirrored with a significant 0.8 bed day saving per person in the external evaluation [20]. Resource utilization outcome measures and the actual rate of savings will be the subject of further evaluation.

This summative evaluation was conducted in a living health system as the first phase of a government funding initiative to move from activity to value-based funding when Monash Health services were under significant funding constraints. Success was achieved in the real world without going through the traditional research route with a trial before rolling out an implementation. The positive feature of this approach is that (to date) it has not gone the way of many beautifully designed and executed pilots that never achieved implementation. In the first phase, the successful delivery of care was within existing funding. The MonashWatch-type model deployment in other health services in the second and third phases has the opportunity to improve on trial methodology. The addition of more research resources, given the current successful proof of concept, would enable the conduct of a more sophisticated randomized propensity-matched trial.

Ongoing study of the data is needed to identify who benefits from which components and how the intervention can be improved for different groups. There is a need to continue to shift current care pathways and health systems to adapt care to the needs of vulnerable MonashWatch-type patients. A whole of systems transformation is needed to respond to the dynamics of unstable health journeys, beyond the current single disease or condition siloed care. Outcome-based funding has the potential to make major inroads into fragmented care. Macrolevel health service funding changes in the Australian Health System such as in northern Spain [29] would be a great advantage to improve integrated care but is unlikely due to political barriers.

### Conclusion

The MonashWatch telehealth and coaching intervention using the HLCC innovative funding model was effective in a local catchment area of a hospital in a highly disadvantaged community and achieved its health service funding model objectives. It requires ongoing and broader implementation and evaluation. In the future, evaluation of additional teams is needed to confirm these findings in different populations and settings. Two additional teams are now in place. Ultimately, the progressive scaling up to a multisite intervention will require ongoing tailoring and evaluation with feedback for improvement.

## Appendix

Multimedia Appendix 1 CONSORT-EHEALTH checklist (V 1.6.1).

## Abbreviations

AIHW: Australian Institute of Health and Welfare
ANCOVA: analysis of covariance
DHHS: Department of Health and Human Services
HLCC: HealthLinks: Chronic Care
ICD: International Statistical Classification of Diseases
